# Hemodynamic and biological correlates of glomerular hyperfiltration in sickle cell patients before and under renin–angiotensin system blocker

**DOI:** 10.1038/s41598-021-91161-y

**Published:** 2021-06-03

**Authors:** Jean-Philippe Haymann, Nadjib Hammoudi, Marine Livrozet, Aline Santin, Sarah Mattioni, Emmanuel Letavernier, Vincent Frochot, Camille Saint Jacques, Olivier Steichen, Gilles Grateau, Michel Chaignon, François Lionnet

**Affiliations:** 1 Service d’Explorations Fonctionnelles Multidisciplinaires, AP-HP, Hôpital Tenon, Sorbonne Université, 75020 Paris, France; 2 INSERM, UMR_S 1155, AP-HP, Hôpital Tenon, Sorbonne Université, 75020 Paris, France; 3 INSERM, Institute of Cardiometabolism and Nutrition (ICAN), ACTION Study Group and Department of Cardiology, Institute of Cardiology (AP-HP), Hôpital Pitié-Salpêtrière, Sorbonne Université, Boulevard de l’hôpital, 75013 Paris, France; 4 Department of Internal Medicine, Centre de reference de la drepanocytose (AP-HP), Centre Hospitalier Universitaire Tenon, Sorbonne Université, rue de la Chine, 75020 Paris, France

**Keywords:** Physiology, Nephrology

## Abstract

Glomerular hyperfiltration alone or associated with albuminuria is a well-known feature of sickle cell associated nephropathy. Though, glomerular hyperfiltration is currently considered to be related to a high renal plasma flow and chronic hemolysis, cardiac output influence on measured glomerular filtration rate (mGFR) have not been investigated so far. Thirty seven homozygous sickle cell patients (SCA) from the RAND study investigated before and under angiotensin converting enzyme inhibitor (ACEI) were included. Both mGFR and cardiac index (CI) were high (> 110 ml/min/1.73 m^2^ and > 3.5 l/m^2^ in 81% and 97% of cases) with low systemic vascular resistance (SVR) (< 700 dynes/s/cm^−5^) in 38% of cases. mGFR association with CI and SVR were significant at baseline (respectively ρ: 0.44, p = 0.008 and ρ: − 0.37, p = 0.02) and under ACEI (p = 0.007 and 0.01 respectively), in accordance with previous data showing that hyperfiltration was linked to an increased glomerular perfusion and a glomerulomegaly rather than increased capillary hydrostatic pressure. Of notice, after adjustment on CI, mGFR remained associated with reticulocyte count and albuminuria under ACEI (p = 0.006 and 0.02 respectively). Our results suggest that hyperfiltration is tightly linked to an increased cardiac output which may account for an increased renal blood flow. Chronic hemolysis could be a relevant factor accounting for hyperfiltration potentially acting on glomerular enlargement which appears as a key factor. Our data suggest that cardiac output assessment is a relevant tool in the routine management and monitoring of SCA nephropathy.

## Introduction

Previous studies reported a very high prevalence of hyperfiltration in young sickle cell anemia (SCA) patients with up to 60–80% in children and 50–60% in young adults^1–3^. Sickle cell anemia nephropathy may also associate micro and macroalbuminuria and ultimately renal failure^3–9^, though the natural history of SCA nephropathy is poorly understood. Nevertheless, this early event, i.e. increased measured glomerular filtration rate (mGFR) was investigated in small but well designed studies^3,10^ suggesting that an unusual high renal plasma flow altogether with a low, but sometimes normal filtration fraction was at play^10,11^. Though cardiac output is increased in most SCA patients^12^ altogether with a low blood pressure^13^ suggesting low systemic vascular resistance (SVR), to our knowledge, cardiovascular parameters associated with mGFR have not been studied so far. We took advantage of our previous RAND trial^14^ showing a beneficial effect of a 6 months angiotensin converting enzyme inhibitor (ACEI) treatment on macroalbuminuria in adult SCA patients at the time of hyperfiltration to study mGFR correlates with hemodynamic and biological parameters before and under renin-angiotensin system blocker, i.e. presumably in the setting of a normal or low filtration fraction. Our results suggest that mGFR is tighly correlated to cardiac output suggesting an impairment of renal hemodynamic autoregulation.

## Results

As shown Table 1, among 14 male and 23 female homozygous sickle cell patients, median age was 30.9 ± 7.4 years old with 5 (14%) overweight patients with a maximum value of 30.2 kg/m^2^. Thirty three percent of patients were under a long term hydroxycarbamide treatment and 17% with a history of blood transfusions. All patients were normotensive at baseline with a maximum value of 129 and 75 mmHg for systolic and diastolic blood pressure respectively. Pulse pressure (PP) and Pulse Wave Velocity (PWV) median values were 52.8 [49.0–59.0] mmHg and 8.0 [7.1–8.6]m/s respectively. Cardiac index (CI) was high, above 3.5 and 4.5 l/min/m^2^ in 97% and 51% of patients respectively, and SVR values below normal range (700 dynes/s/cm^−5^) in 14 cases (38%) with no patients with high SVR values (most below 1000 dynes/s/cm^−5^). Of notice, CI and SVR did not change under ACEI treatment (4.62 versus 4.66 ml/min/m^2^ (p = 0.36) and 759 versus 738 dynes/s/cm^−5^ (p = 0.28) respectively). TRV values were available in 26 patients and above 2.5 m/s in 46% of cases. Mean mGFR assessed by ^51^Cr EDTA renal clearance was 131 ml/min/1.73 m^2^ before initiation of ACEI (Table 2), with a mean serum creatinine of 46.6 µmol/l (Table 1). Thus hyperfiltration (assessed by mGFR > 110 ml/min/1.73 m^2^) was present in 81% of cases (n = 30) with no significant variation under ACEI treatment whereas DBP and MAP decreased significantly from 57.3 to 54.2 mmHg and 75.2 to 72.8 mmHg (p = 0.02 and p = 0.04 respectively). Before initiation of ACEI, microabuminuria and macroalbuminuria were detected in 48.6% and 43.2% of cases respectively with ACR < 5 mg/mmol only in two patients. Anemia was present in all patients with a median haemoglobin level of 8.3 [7.7–8.9]g/dl, with a high reticulocyte count, plasma LDH and bilirubin concentrations (Table 1).

**Table 1:** Population characteristics.

Clinical parameters	N = 37
Age (years)	31.1 ± 7.7
BMI (kg/m^2^)	22.4 ± 3.2
Gender (M/F)	14/23
Ulcer (%)	8.1%
ACS (%)	77%
Retinopathy (%)	24%
Priapism (% males)	28.6%
Hydroxycarbamide (%)	35.1%
**Hemodynam ic parameters**	
SBP (mmHg)	111 ± 9.3
DBP (mmHg)	57 ± 7.0
MAP (mmHg)	75 ± 7.1
HR (beats/min)	73 ± 9.7
PP (mmHg)	53 ± 7.0
PW V (m/s)	8.0 ± 1.2
CI (l/min/m^2^)	4.6 ± 0.7
LVMI (g/m^2^)	110 ± 21.1
TRV (m/s)	2.50 ± 0.18
SVR (dynes/s/cm^−5^)	759 ± 151
**Biological parameters**	
Creatinine (µmol/l)	46.6 ± 12.3
ACR (mg/mmol)	47.8 ± 52.0
Hb (g/dl)	8.4 ± 1.1
Reticulocyte (10^3^/mm^3^)	303 ± 141
Bilirubin (UI/l)	65 ± 42
LDH (UI/l)	478 ± 122
ADMA (µmol/l)	1.07 ± 0.45
Fasting glucose (mmol/l)	4.90 ± 0.43
Fasting insulin (mUI/l)	4.58 ± 2.7

BMI: body mass index; ACS: acute chest syndrome; SBP: systolic blood pressure; DBP: diastolic blood pressure; MAP: mean arterial pressure; HR: heart rate; PP: pulse pressure; PWV: pulse wave velocity; CI: cardiac index; LVMI: left ventricular mass index; TRV: tricuspid regurgitation velocity; SVR: systemic vascular resistance; ACR: albumin creatinine ratio; Hb: haemoglobin; LDH: lactic dehydrogenase; ADMA: asymetric dimethyl arginine.

**Table 2:** Comparison of mGFR and eGFR values before and under ACEI treatment.

	Basal (N = 37)	Under RAS blocker (N = 37)	P
**mGFR**			
Renal ^51^CrEDTA clearance	131 ± 21	128 ± 23	0.26
Plasma ^51^CrEDTA clearance	127 ± 19	124 ± 17	0.08
Creatinine urinary clearance	182 ± 37	192 ± 49	0.35
**eGFR**			
CKD EPI equation	156 ± 15	159 ± 16	0.35
MDRD equation	190 ± 49	190 ± 50	0.98
Cockcroft equation	192 ± 45	191 ± 51	0.75

mGFR values, CKD EPI and MDRD equations are expressed in ml/min/1.73 m^2^. Cockcroft equation is expressed in ml/min.

**Figure 1:**
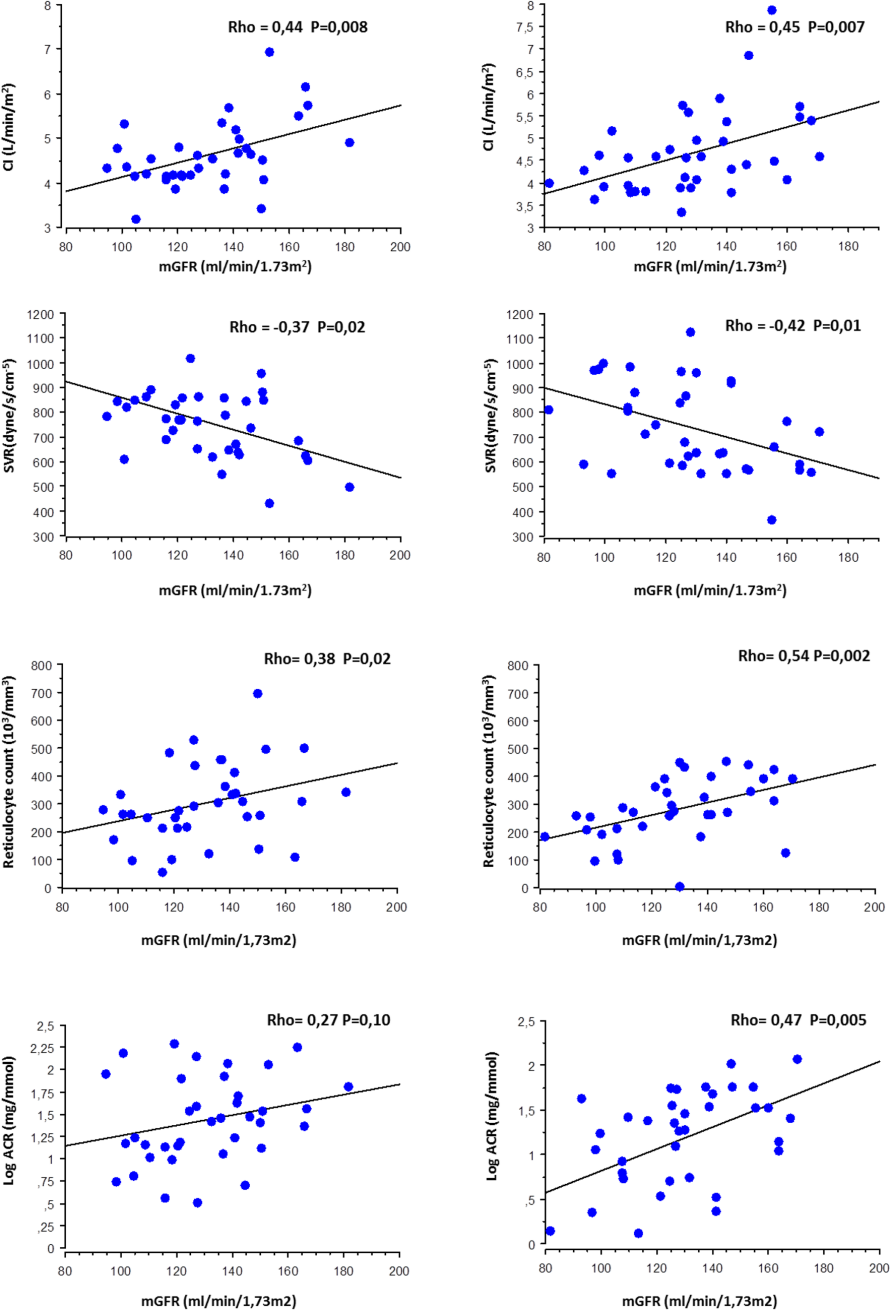
mGFR association with CI, SVR, reticulocyte count and ACR before and under ACEI treatment. CI: cardiac index; SVR: systemic vascular resistance; ACR: albumin creatinine ratio.

As shown Table 3 and Fig. 1, mGFR was significantly associated with CI and SVR at baseline: high mGFR values were associated with high CI (rho = 0.44, p = 0.008) and low SVR (rho = − 0.37, p = 0.02). Of notice, under ACEI, mGFR association with CI and SVR was even stronger. Moreover, in this latter case, mGFR was also associated with some additional parameters such as TRV (rho = 0.40, p = 0.04) but also reticulocyte count (rho = 0.54, p = 0.002) and ACR (rho = 0.47, p = 0.005). However, as shown Table 4, ACR and reticulocyte count remained significant after adjustment either for CI or SVR.

**Table 3:** mGFR correlates before and under RAS blockade.

	Basal		Under RAS blocker	
	Rho	p	Rho	p
Age (years)	− 0.1	0.95	− 0.15	0.37
BMI (kg/m^2^)	− 0.08	0.61	0.02	0.98
ACR (mg/mmol)	0.27	0.10	0.47	0.005
CI (l/min/m^2^)	0.44	0.008	0.45	0.007
TRV (m/s)	0.09	0.63	0.40	0.04
SVR (dynes/s/cm^−5^)	− 0.37	0.03	− 0.42	0.01
Hb (g/dl)	− 0.69	0.49	− 0.08	0.61
Reticulocyte (10^3^/mm^3^)	0.38	0.02	0.54	0.002
Bilirubin (UI/l)	0.05	0.76	0.14	0.4
LDH (UI/l)	− 0.20	0.23	− 0.13	0.45
HbF (%)	− 0.23	0.16	− 0.3	0.08
SBP (mmHg)	− 0.01	0.96	− 0.06	0.73
DBP (mmHg)	− 0.17	0.29	− 0.30	0.07
PAM (mmHg)	− 0.16	0.33	− 0.21	0.21
PWV (m/s)	− 0.26	0.12	− 0.24	0.16
PP (mmHg)	0.23	0.18	0.18	0.27
ADMA (µmol/l)	− 0.04	0.81	0.09	0.58

BMI: body mass index; ACS: acute chest syndrome; SBP: Systolic blood pressure; DBP: diastolic blood pressure; MAP: mean arterial pressure; HR: heart rate; PP: pulse pressure; PWV: pulse wave velocity; CI: cardiac index; LVMI: left ventricular mass index; TRV: tricuspid regurgitation velocity; SVR: systemic vascular resistance; ACR: albumin creatinine ratio; Hb: haemoglobin; LDH: lactic dehydrogenase.

**Table 4:** mGFR association with different parameters under ACEI after adjustment with CI (model 1) or SVR (model 2).

	Under RAS blockers		Under RAS blockers	
	Model 1		Model 2	
	b	p	b	p
CI (l/min/m^2^)	–	–	0.31	0.18
SVR (dynes/s/cm−^5^)	− 0.20	0.39	–	–
TRV (m/s)	0.22	0.28	0.22	0.21
Age (years)	− 0.26	0.09	− 0.09	0.58
BMI (kg/m^2^)	0.01	0.94	− 0.10	0.50
Log ACR (mg/mmol)	0.37	0.02	0.36	0.02
SBP (mmHg)	− 0.10	0.51	0.06	0.69
DBP (mmHg)	− 0.27	0.17	0.06	0.78
PAM (mmHg)	− 0.21	0 .17	0.06	0.71
PWV (m/s)	− 0.23	0.16	− 0.24	0.13
PP (mmHg)	0.02	0.90	0.05	0.73
Hb (%)	0.01	0.96	0.05	0.72
Reticulocyte (103/mm^3^)	0.41	0.006	0.39	0.01
Bilirubin (UI/l)	− 0.06	0.69	− 0.12	0.46
LDH (UI/l)	− 0.20	0.20	− 0.24	0.13
HbF (%)	− 0.24	0.12	− 0.24	0.16
ADMA (µmol/l)	− 0.01	0.97	0.01	0.93

## Discussion

Our results show that in our SCA population with glomerular hyperfiltration, mGFR is strongly associated with cardiac output which is increased in most patients altogether with a low SVR in 38% of cases. Of notice, SVR was normal (but not high) in all other cases with no blood hypertension reported despite the presence of albuminuria, thus raising the issue of the pathophysiological process of glomerular hyperfiltration. It has been suggested that glomerular hyperfiltration is driven by increased glomerular perfusion and increased effective glomerular filtration surface area (glomerular enlargement) rather than by increased glomerular capillary hydrostatic pressure, which would separate this condition from early diabetic nephropathy^15–17^. Indeed, a high cardiac output and reduced peripheral vascular resistance was previously reported in six adult SCA patients with mean CI and SVR values in the same range than our population (5.0 l/min/m^2^ and 856 dynes/s/cm^−5^ respectively), however renal function was not reported^18^. Our findings that SVR are below 1000 dynes/s/cm^−5^ in all cases and low in 40% of patients suggest that renal vascular resistance may also be low. Indeed, an increased renal plasma flow was reported in several studies presumably related to an increased cardiac output though no data were available^10,11,19^. However, renal hemodynamic regulation is expected to increase afferent arteriolar tone in order to counterbalance an increased renal plasma flow^20^ and thus maintain a GFR within normal values. We may thus speculate that this regulation may be less efficient or possibly impaired in some individuals. Though we cannot fully address the issue of an impaired tubular glomerular feedback, we can rule out in most cases an increased glomerular capillary hydrostatic pressure responsible for hyperfiltration as (1) a low/normal filtration fraction was reported in previous studies^10,11^; (2) mGFR was similar before and under ACEI thus pointing out increased renal blood flow (rather than pressure) as a key determinant of hyperfiltration in our patients. We thus speculate that in most SCA patients with glomerular hyperfiltration, filtration occurs along the whole capillary surface due to increased renal plasma flow even in a normal hydrostatic pressure setting (i.e. no renal functional reserve). Thus according to this view, normalizing (under ACEI) hydrostatic pressure in a SCA patient would have no short term effect on GFR conversely to ACR which should decrease.

Alternatively, hemodynamic autoregulation may be preserved and increased effective glomerular filtration surface alone (in the setting of increased glomerular perfusion) may account for mGFR increase by 80% in some cases. Indeed, a glomerulomegaly responsible for an increased glomerular surface/volume and filtration coefficient (Kf) is reported to be a hallmark of SCA nephropathy^10,11,21–26^. This increased glomerular growth can be observed at a time of hyperfiltration as early as in infancy^1,17^. Altogether, glomerular hyperfiltration in SCA looks very similar to glomerular hyperfiltration in pregnancy where it is the consequence of increased cardiac output and decreased renal afferent and efferent arteriolar resistance responsible for an increased renal blood flow with glomerular enlargement and no increased filtration fraction^16^. However, the current view is that relaxin, a pregnancy hormone, which reduces myogenic reactivity of small renal arteries^27^ is responsible for glomerular hyperfiltration which is not the case in SCA patients, where the basis for renal hyperperfusion remains under debate. Indeed, such hyperperfusion cannot be readily ascribed to anaemia or increased plasma volume because correction of anaemia by HbA-containing RBC transfusions does not reverse hyperperfusion^28^ and chronic anaemia from causes other than SCA is not usually associated with renal hyperperfusion^17,29^. Among other causal factors, chronic hemolysis noteworthy through haem oxygenase–carbon monoxide (HO–CO) system is pointed out as induction of HO–CO might contribute to regional hyperperfusion of the kidney and other vascular beds^17^. Our previous findings showing that different markers of chronic hemolysis including reticulocyte count were independent risk factors for glomerular hyperfiltration in a SCA population^2^ are strengthened in this study showing that mGFR is associated with reticulocyte count even after adjustment for cardiac index or SVR in SCA patients under ACEI. Though there may be more than one mechanism by which greater rates of chronic hemolysis could contribute to hyperfiltration in the kidney, our data suggests that chronic hemolysis may be linked to glomerulomegaly dependent hyperfiltration process. According to this view, the reported beneficial effect of hydroxyurea treatment at decreasing hyperfiltration in children^30^ could be explained either by a sustained improvement of anemia decreasing cardiac output but also a beneficial effect on glomerular remodeling, i.e. glomerulomegaly. We speculate that a glomerular remodeling slowdown could then be an interesting therapeutical target.

In addition to hyperfiltration, glomerulomegaly accounts also for the reported increased glomerular permselectivity assessed by fractional clearances of dextran^10^, and the high prevalence of albuminuria in SCA patients^2–6^. Though an increased glomerular hydraulic pressure (ΔP) is likely to occur at least in macroalbuminuric patients, as a decreased albuminuria under ACEI treatment was detected in this subgroup^14^, our data showing that mGFR was associated with albuminuria only in patients under ACEI suggests that glomerular remodeling independently of ΔP may account for increased glomerular permeability to albuminuria, presumably through filtration slits stretching. According to this view, ACR values under ACEI would be an interesting bed-side relevant biomarker of glomerulomegaly, i.e. of renal structural remodelling in this population.

Limitation of the study: our data show that hyperfiltration is related to an increased cardiac output, thus leading presumably to a high renal plasma flow. The issue whether in our population, renal vascular resistance would be low as previously reported in SCA patients^10^ or normal^11^ is unfortunately unknown as renal plasma flow measurement was unavailable for this study. We speculate that alike systemic vascular resistance, low renal vascular resistance may occur in some patients accounting for unusual high mGFR values in the setting of a high cardiac output. Moreover, though we cannot exclude a further decrease renal plasma flow under ACEI, it seems unlikely as CI and SVR did not change. The complex pathological cascades of chronic hemolysis metabolites interplay with cardiovascular/ renal hemodynamics and the relevant determinants of glomerulomegaly responsible for hyperfiltration are obviously beyond the scope of the present study and require to be specifically addressed.

To conclude, our results suggest that hyperfiltration is tightly linked to an increased cardiac output and low systemic vascular resistance which may account for an increased blood flow both in kidneys and likely also in most other organs. These findings stress glomerular enlargement as a likely key step in the history of hyperfiltration and point out cardiac output assessment as a relevant tool in the routine management and monitoring of SCA nephropathy.

## Material and methods

### Ethics statement

All patients signed written informed consents before inclusion in the study. The Regression de l’Albuminurie dans la Nephropathie Drepanocytaire (RAND) study design was approved by the local ethics committee (Direction Générale pour la recherche et l'innovation. Comité Consultatif sur le traitement de l'information en matière de recherche dans le domaine de la santé, DGRI CCTIRS MG/CP09.503, 9 July 2009) and registered at ClinicalTrials.gov (NCT01195818**).** All methods were performed in accordance with the relevant guidelines and regulations.

### Patients and study design

The RAND study is a prospective multicentric non-randomized trial that took place between September 2010 and September 2014 aimed at studying as a main objective the effect of ACEI treatment on albuminuria after a 6 month-trial (Supplementary information). Forty-two adult patients with sickle cell disease were enrolled and evaluated at month 6, as described in Fig. 1 and reported previously^14^. Thirty seven patients underwent a complete cardiac evaluation at baseline and under ACEI at 6 months and were thus included in the present study (Tricuspid regurgitation velocity (TRV) was available only in 26 patients). Eligible criteria were adult homozygous SS patients aged > 18 years, with a minimum urine albumin:creatinine ratio (ACR) > 10 mg/mmol creatinine on two different random urine spots performed at least 1 month after any acute SCA complication, and a recent estimated GFR (eGFR; calculated using the Modification of Diet in Renal Disease [MDRD] Study equation) > 130 ml/min/1.73 m^2^. Exclusion criteria were acute SCA complication within 1 month prior to study inclusion, patients receiving blood transfusions within a 3-month period or initiation of hydroxycarbamide within 3 months, ongoing pregnancy or treatment with nonsteroidal anti-inflammatory drugs, lithium or antihypertensive agents, human immunodeficiency virus or hepatitis C infection, known allergic reactions to ACEIs or angiotensin II receptor blockers, hereditary or idiopathic angio-oedema, galactosemia or lactase deficiency. Patients were included for 7 months: ramipril was started at 2.5 mg/day after baseline evaluation (Visit 1, V1), increased to 5 mg/day after Visit 2 (V2) 1 month later; treatment was stopped at month 6 following a complete evaluation on Visit 3 (V3). In case of side effects, ramipril was replaced by irbesartan (75 mg up to V2 and then 150 mg/day up to V3). Complete renal and cardiovascular evaluation including measured GFR (mGFR) and doppler echocardiography were performed at baseline and month 6th. ACR ≤ 3 mg/mmol creatinine was considered as normoalbuminuria, microalbuminuria was defined by an ACR of 3–30 mg/mmol creatinine and macroalbuminuria by an ACR > 30 mg/mmol creatinine. mGFR was assessed by ^51^Cr-EDTA renal clearance, as described previously^3^. Briefly, 1.8–3.5 MBq of ^51^Cr-EDTA (GE Healthcare, Velizy, France) was injected intravenously as a single bolus. Average urinary ^51^Cr-EDTA clearance, average plasmatic ^51^Cr EDTA and urinary creatinine clearance were determined during five to six consecutive 30-min clearance periods. GFR measurements were average and standardized for body surface area (1.73 m^2^). Hyperfiltration was defined above a threshold value of 110 ml/min/1.73 m^2^ for mGFR and renal insufficiency was defined as mGFR < 60 ml/ min/1.73 m^2^.

### Cardiovascular evaluation

Patients were examined in a quiet, temperature-controlled room. Blood pressure was measured after 15 min of rest in a supine position with an oscillometric method and a cuff of appropriate size. The average of five consecutive measurements was calculated. Aortic stiffness was measured through the carotid to femoral pulsewave velocity (PWV) between the two sites by the foot-to-foot velocity method (Complior; ALAM Medical, Pantin, France), as described previously and validated^31,32^. Briefly, PWV was calculated from measurements of pulse transit time and the distance traveled by the pulse between two recording sites: pulse wave velocity distance (m)/transit time (seconds). It expresses the elastic properties of the descending and abdominal aorta and the iliofemoral segments. PWV was defined as the mean of four determinations. Doppler echocardiography examinations were performed by an experienced physician according to the guidelines^33^. All the examinations were analyzed off-line blinded to study time point, to clinical and biological data. From M mode, the following measurements were made at end diastole: left ventricular (LV) internal diameter, interventricular, septal and posterior wall thicknesses. LV mass was derived and indexed to body surface area^33^. From 2D mode, LV volumes and ejection fraction (LVEF) were derived from Simpson’s modified biplane method. Left atrial volume was also measured and indexed to body surface area. From pulsed wave Doppler mode, LV outflow tract time–velocity integral and early peak diastolic velocity of the mitral (E) inflow were measured. Cardiac output was calculated and indexed (CI) to body surface area as recommended^34,35^. From continuous wave Doppler, peak tricuspid regurgitation was recorded in multiple views and the highest level of velocity was selected. Elevated pulmonary systolic pressure was defined by a peak tricuspid regurgitation velocity (TRV) > 2.5 m/s^36^. Systemic Vascular Resistance (SVR) was calculated in units of dyn s cm^−5^ as: 80 × Mean Arterial Pressure/Cardiac Output (normal range 700–1600 dyn s cm^−5^).

### Assays

As previously described assays were performed in our lab^2,14^. Serum and urinary creatinine levels were measured by the enzymatic method on a Konelab 20 analyzer from Thermo Fisher Scientific. Uric acid levels were measured with the Konelab analyzer. Total CO_2_ in blood, ionized calcium, sodium, and potassium levels were measured with an ABL 815 from Radiometer. Calcium and magnesium serum and urinary levels were measured with the PerkinElmer 3300 atomic absorption spectrometer. Plasma Aldosterone was measured by radioimmunoassay kits (Immunodiagnostics Systems Ltd.) Plasma asymmetric dimethyl arginine (ADMA), symmetric dimethyl arginine (SDMA) and arginin were measured by a high liquid performance chromatography coupled with fluorimeter detection. Other laboratory values were measured using standard hospital laboratory techniques.

### Statistical analyses

Variables were expressed as percentages, means ± SD, or medians (IQR: interquartile range), as appropriate. Comparison of biological and hemodynamic data measured at different visits was tested with a non parametric Wilcoxon rank test for quantitative variables. Association between mGFR and variables of interest were tested separately using Spearman correlation test and a multilinear regression analysis when adjustment on CI or SVR was performed. A two-sided P-value < 0.05 indicated statistical significance. Statistical analyses were performed with Statview 5.0.

## Supplementary Information

Supplementary Information.
