# Supplementary material for: Hemodynamic and biological correlates of glomerular hyperfiltration in sickle cell patients before and under renin–angiotensin system blocker
Source: Sci Rep. 2021 Jun 3;11:11682. doi: 10.1038/s41598-021-91161-y (PMC8175337; doi:10.1038/s41598-021-91161-y)
Supplement: Supplementary file 1 — Supplementary Information. [file 41598_2021_91161_MOESM1_ESM.docx]

**Glomerular hyperfiltration: hemodynamic and biological correlates in sickle cell patients before and under renin-angiotensin system blocker**

Jean-Philippe Haymann^1,2^ MD, PhD, Nadjib Hammoudi^3^ MD, PhD, Marine Livrozet^1^ MD, PhD, Aline Santin^4^ MD, Sarah Mattioni^4^ MD, Emmanuel Letavernier^1,2^  MD, PhD, Vincent Frochot^1,2^  MD, PhD, Camille Saint Jacques^1^ MD, Olivier Steichen^4^ MD, PhD, Gilles Grateau^4^ MD, Michel Chaignon^1^ MD, François Lionnet^4^ MD.

1*.* Sorbonne Université, Service d’Explorations Fonctionnelles Multidisciplinaires, AP-HP, Hôpital Tenon, Paris 75020, France.

2. Sorbonne Université, INSERM, UMR_S 1155, AP-HP, Hôpital Tenon, Paris 75020, France

3*.*  Sorbonne Université, Inserm, Institute of Cardiometabolism and Nutrition (ICAN), ACTION Study Group and Department of Cardiology, Institute of Cardiology (AP-HP), Hôpital Pitié- Salpêtrière, Boulevard de l'hôpital, Paris F-75013, France.

4. Sorbonne Université, Department of Internal Medicine, centre de référence de la drépanocytose (AP-HP), Centre Hospitalier Universitaire Tenon, rue de la Chine, 75020 Paris, France.

***** Correspondence: jean-philippe.haymann@aphp.fr ; Tel.: +33-156-016-774; Fax: +33-156-017-003

**Trial Protocol :**

**https;//clinicaltrials.gov/ct2/show/record/NCT01195818?recrs=e&cond=sickle+cell+disease&cntry=FR&city=Paris&draw=4&rank=6**

Tracking Information

First Submitted Date ICMJE September 3, 2010

First Posted Date ICMJE September 6, 2010

Last Update Posted Date June 18, 2015

Study Start Date ICMJE September 2010

Actual Primary Completion Date: September 2014 (Final data collection date for primary outcome measure)

**Current Primary Outcome Measures ICMJE** (submitted: February 15, 2011) Comparison of albuminuria/ urinary creatinin ratio before and after a 6 months RAS inhibitor treatment period [ Time Frame: 6 months RAS inhibitor treatment period ]

**Original Primary Outcome Measures ICMJE (submitted: September 3, 2010):**

Comparison of albuminuria/ urinary creatinine ratio before and after a 6 months RAS inhibitor treatment period [ Time Frame: 6 months RAS inhibitor treatment period ]

Change History Complete list of historical versions of study NCT01195818 on ClinicalTrials.gov Archive Site

**Current Secondary Outcome Measures ICMJE (submitted: February 15, 2011)**

Comparison of albuminuria/ urinary creatinine ratio under RAS inhibitor treatment and after 1 month wash-out period [ Time Frame: 1 month wash-out period ]

Comparison of glomerular Filtration Rate (51CR EDTA clearance) before and after a 6 months RAS inhibitor treatment period [ Time Frame: 6 months RAS inhibitor treatment period ]

Study of at base line and under RAS treatment : 1) biomarkers evaluating NO metabolism (ADMA, arginase....), endothelial dysfunction (VEGF, PLGF and endothelin 1), hemolysis (LDH, haemoglobin, heme...) 2) heart and vessels ( cardiac doppler, aortic pulse wave velocity and microvascular brachial laser-Doppler,)

**Original Secondary Outcome Measures ICMJE (submitted: September 3, 2010)**

Comparison of albuminuria/ urinary créatinine ratio under RAS inhibitor treatment and after 1 month wash-out period [ Time Frame: 1 month wash-out period ]

Comparison of glomerular Filtration Rate (51CR EDTA clearance) before and after a 6 months RAS inhibitor treatment period [ Time Frame: 6 months RAS inhibitor treatment period ]

Study of at base line and under RAS treatment : 1) biomarkers evaluating NO metabolism (ADMA, arginase....), endothelial dysfunction (VEGF, PLGF and endothelin 1), hemolysis (LDH, haemoglobin, heme...) 2) heart and vessels ( cardiac doppler, aortic pulse wave velocity and microvascular brachial laser-Doppler,)

Current Other Pre-specified Outcome Measures Not Provided

Original Other Pre-specified Outcome Measures Not Provided

**Descriptive Information**

**Brief Title ICMJE: Albuminuria Reduction With Renin Angiotensin System Inhibitors in SCA Patients**

**Official Title ICMJE: Effect of RAS Inhibitors on Albuminuria, Hyperfiltration and Endothelial Dysfunction in a Sickle Cell Disease Population.**

**Brief Summary:** The prevalence of Sickle Cell Associated Nephropathy (SCAN) is increasing and is a growing concern. Microalbuminuria is detected in the early onset of SCAN. Noteworthy, as in diabetic nephropathy, hyperfiltration seems to be a frequent finding, with, in our series, an overall incidence of 57 % and suggests a pathological links between glomerular hyperpressure and glomerulosclerosis which occurs several years after. Nitric oxide (NO) deficiency and the renin angiotensin system (RAS) are likely to be involved in the glomerular hyperpressure leading to hyperfiltration. Renin angiotensin antagonists are currently given for NEPHROPROTECTION in numerous nephropathy including SCAN despite few available reports. The percentage of decrease of albuminuria or the percentage of responders (ie patient normalizing albuminuria) has never been reported to our knowledge in SCAN patients at the time of hyperfiltration. The focus of our study is therefore to 1) Quantify albuminuria reduction after 6 months RAS treatment (primary end point); 2) Quantify glomerular filtration rate (GFR) reduction after 6 months of RAS treatment, and to test the hypothesis of a beneficial effect of RAS inhibitors on several biomarkers assessing hemolysis, NO inhibition and the endothelial damages (secondary end points). The ultimate aim of our study is to identify relevant (new) biomarkers associated to hyperfiltration and/or albuminuria decrease (/normalization).

**Detailed Description:**

This is a non randomized prospective multicentered study testing the effect of RAS treatment with a wash out period at the end of the study.

Enrollment and informed consent will be performed in three AP-HP centers. Visit n° 1 will be performed in Hospital Tenon Center ( FONCTIONNEL Exploration Service) where final eligibility will be followed by several investigations aiming to measure albuminuria, basal GFR (51 cr EDTA clearance), cardiac parameters (doppler study); aortic stiffness (aortic pulse wave velocity) and endothelial dysfunction (microvascular laser-doppler, and blood and urine sample for assessment of several biomarkers).

At the end of the evaluation, Ramipril administration will be initiated (for at least six months).

Tolerance will be check up at visit n°2 (month 1) (clinical examination) and at the visit n°3 (month 6 ) (clinical examination). Patients will be contacted by the investigators every two month between each visit in order to evaluate tolerance. In case of cough with Ramipril, the treatment may be change by Irbesartan. Posology of treatment may be reduced in case of intolerance.

Treatment full dose (Ramipril 5mg/day or Irbesartan (300mg/day) will be obtained at the visit 2 and stopped at the end of visit 3:

Assessment of RAS treatment effect on albuminuria, GFR, heart, aorta and microvessels will be performed at visit 3 (under RAS treatment) with the same procedure as visit 1.

Visit 4 will be performed after a 1 month wash out period in order to check whether the expected reduction of albuminuria under RAS treatment is sustained or not.

**Study Type ICMJE: Interventional**

Study Phase ICMJE Not Applicable

Study Design ICMJE Allocation: N/A

Intervention Model: Single Group Assignment

Masking: None (Open Label)

Primary Purpose: Prevention

Condition ICMJE: Sickle Cell Disease

Intervention ICMJE: Drug: RAS Inhibitors

Clinical trial testing the effect of RAS inhibitors (Ramipril or Irbesartan)on sickle cell disease patients wit hyperfiltration and albuminuria for a six month period followed by one month wash out period. Drug will be given after the first GFR measurement on a daily basis with a full dose given after the first month.

Study Arms ICMJE: Experimental: RAS Inhibitors

Intervention: Drug: RAS Inhibitors

**Publications ***

Dharnidharka VR, Dabbagh S, Atiyeh B, Simpson P, Sarnaik S. Prevalence of microalbuminuria in children with sickle cell disease. Pediatr Nephrol. 1998 Aug;12(6):475-8.

Guasch A, Navarrete J, Nass K, Zayas CF. Glomerular involvement in adults with sickle cell hemoglobinopathies: Prevalence and clinical correlates of progressive renal failure. J Am Soc Nephrol. 2006 Aug;17(8):2228-35. Epub 2006 Jul 12.

Falk RJ, Scheinman J, Phillips G, Orringer E, Johnson A, Jennette JC. Prevalence and pathologic features of sickle cell nephropathy and response to inhibition of angiotensin-converting enzyme. N Engl J Med. 1992 Apr 2;326(14):910-5.

Olson JL, Hostetter TH, Rennke HG, Brenner BM, Venkatachalam MA. Altered glomerular permselectivity and progressive sclerosis following extreme ablation of renal mass. Kidney Int. 1982 Aug;22(2):112-26.

Serjeant GR, Higgs DR, Hambleton IR. Elderly survivors with homozygous sickle cell disease. N Engl J Med. 2007 Feb 8;356(6):642-3.

Manci EA, Culberson DE, Yang YM, Gardner TM, Powell R, Haynes J Jr, Shah AK, Mankad VN; Investigators of the Cooperative Study of Sickle Cell Disease. Causes of death in sickle cell disease: an autopsy study. Br J Haematol. 2003 Oct;123(2):359-65.

Powars DR, Elliott-Mills DD, Chan L, Niland J, Hiti AL, Opas LM, Johnson C. Chronic renal failure in sickle cell disease: risk factors, clinical course, and mortality. Ann Intern Med. 1991 Oct 15;115(8):614-20.

Barros FB, Lima CS, Santos AO, Mazo-Ruiz MF, Lima MC, Etchebehere EC, Costa FF, Saad ST, Camargo EE, Ramos CD. 51Cr-EDTA measurements of the glomerular filtration rate in patients with sickle cell anaemia and minor renal damage. Nucl Med Commun. 2006 Dec;27(12):959-62.

Marouf R, Mojiminiyi O, Abdella N, Kortom M, Al Wazzan H. Comparison of renal function markers in Kuwaiti patients with sickle cell disease. J Clin Pathol. 2006 Apr;59(4):345-51.

Vedel P, Obel J, Nielsen FS, Bang LE, Svendsen TL, Pedersen OB, Parving HH. Glomerular hyperfiltration in microalbuminuric NIDDM patients. Diabetologia. 1996 Dec;39(12):1584-9.

Berg UB. Differences in decline in GFR with age between males and females. Reference data on clearances of inulin and PAH in potential kidney donors. Nephrol Dial Transplant. 2006 Sep;21(9):2577-82. Epub 2006 May 23.

Chiarelli F, Cipollone F, Romano F, Tumini S, Costantini F, di Ricco L, Pomilio M, Pierdomenico SD, Marini M, Cuccurullo F, Mezzetti A. Increased circulating nitric oxide in young patients with type 1 diabetes and persistent microalbuminuria: relation to glomerular hyperfiltration. Diabetes. 2000 Jul;49(7):1258-63.

Morris CR, Kato GJ, Poljakovic M, Wang X, Blackwelder WC, Sachdev V, Hazen SL, Vichinsky EP, Morris SM Jr, Gladwin MT. Dysregulated arginine metabolism, hemolysis-associated pulmonary hypertension, and mortality in sickle cell disease. JAMA. 2005 Jul 6;294(1):81-90.

Reiter CD, Wang X, Tanus-Santos JE, Hogg N, Cannon RO 3rd, Schechter AN, Gladwin MT. Cell-free hemoglobin limits nitric oxide bioavailability in sickle-cell disease. Nat Med. 2002 Dec;8(12):1383-9. Epub 2002 Nov 11.

Rother RP, Bell L, Hillmen P, Gladwin MT. The clinical sequelae of intravascular hemolysis and extracellular plasma hemoglobin: a novel mechanism of human disease. JAMA. 2005 Apr 6;293(13):1653-62. Review.

Kato GJ, McGowan V, Machado RF, Little JA, Taylor J 6th, Morris CR, Nichols JS, Wang X, Poljakovic M, Morris SM Jr, Gladwin MT. Lactate dehydrogenase as a biomarker of hemolysis-associated nitric oxide resistance, priapism, leg ulceration, pulmonary hypertension, and death in patients with sickle cell disease. Blood. 2006 Mar 15;107(6):2279-85. Epub 2005 Nov 15.

Landburg PP, Teerlink T, Muskiet FA, Duits AJ, Schnog JJ; CURAMA study group. Plasma concentrations of asymmetric dimethylarginine, an endogenous nitric oxide synthase inhibitor, are elevated in sickle cell patients but do not increase further during painful crisis. Am J Hematol. 2008 Jul;83(7):577-9. doi: 10.1002/ajh.21184.

Loutzenhiser R, Bidani A, Chilton L. Renal myogenic response: kinetic attributes and physiological role. Circ Res. 2002 Jun 28;90(12):1316-24.

Sochett EB, Cherney DZ, Curtis JR, Dekker MG, Scholey JW, Miller JA. Impact of renin angiotensin system modulation on the hyperfiltration state in type 1 diabetes. J Am Soc Nephrol. 2006 Jun;17(6):1703-9. Epub 2006 May 3.

Aoki RY, Saad ST. Enalapril reduces the albuminuria of patients with sickle cell disease. Am J Med. 1995 May;98(5):432-5.

Foucan L, Bourhis V, Bangou J, Mérault L, Etienne-Julan M, Salmi RL. A randomized trial of captopril for microalbuminuria in normotensive adults with sickle cell anemia. Am J Med. 1998 Apr;104(4):339-42.

Reboldi G, Gentile G, Angeli F, Verdecchia P. Choice of ACE inhibitor combinations in hypertensive patients with type 2 diabetes: update after recent clinical trials. Vasc Health Risk Manag. 2009;5(1):411-27. Review.

Lionnet F, Arlet JB, Bartolucci P, Habibi A, Ribeil JA, Stankovic K; groupe de recommandations et d'étude de la drépanocytose de l'adulte (GREDA). [Guidelines for management of adult sickle cell disease]. Rev Med Interne. 2009 Sep;30 Suppl 3:S162-223. doi: 10.1016/j.revmed.2009.07.001. Epub 2009 Aug 26. French.

van Deventer HE, George JA, Paiker JE, Becker PJ, Katz IJ. Estimating glomerular filtration rate in black South Africans by use of the modification of diet in renal disease and Cockcroft-Gault equations. Clin Chem. 2008 Jul;54(7):1197-202. doi: 10.1373/clinchem.2007.099085. Epub 2008 May 16.

* Includes publications given by the data provider as well as publications identified by ClinicalTrials.gov Identifier (NCT Number) in Medline.

**Recruitment Information**

Recruitment Status ICMJE: Completed

Actual Enrollment ICMJE (submitted: February 26, 2014): 53

Original Estimated Enrollment ICMJE (submitted: September 3, 2010): 60

Actual Study Completion Date ICMJE September 2014

Actual Primary Completion Date September 2014 (Final data collection date for primary outcome measure)

Eligibility Criteria ICMJE

**Inclusion Criteria:**

Homozygous sickle cell disease

> 18 years

Patient with social insurance

Albuminuria/ urinary creatinin > 10 mg/mmol creatinin (at 2 different times) and MDRD > 140 ml/min/1.73m2.

Written inform consent

**Exclusion Criteria:**

Hemoglobin SC or S-betathalassemia disease

Patient currently treated with: lithium, aspirin, antihypertensive drugs, non steroid-antiinflammatory drugs.

Pregnancy

Woman without contraception

Transfusion within the last 3 months

Intolerance to RAS inhibitors

Treatment with RAS in the last month

Patient with Congenital galactosemia or a malabsorption of glucose or lactase deficiency

Treatment with hydroxyurea began or changed in the last 3 months

Infection with HIV or C hepatitis

Angio-edema

**Sex/Gender ICMJE**

Sexes Eligible for Study: All

Ages ICMJE: 18 Years and older (Adult, Older Adult)

Accepts Healthy Volunteers ICMJE: No

Contacts ICMJE: Contact information is only displayed when the study is recruiting subjects

Listed Location Countries ICMJE: France

Removed Location Countries

**Administrative Information**

**NCT Number ICMJE: NCT01195818**

**Other Study ID Numbers ICMJE: P081110; AOR09063**

Has Data Monitoring Committee No

U.S. FDA-regulated Product Not Provided

IPD Sharing Statement ICMJE Not Provided

**Responsible Party Assistance Publique - Hôpitaux de Paris**

**Study Sponsor ICMJE Assistance Publique - Hôpitaux de Paris**

Collaborators ICMJE Not Provided

Investigators ICMJE Not Provided

PRS Account : Assistance Publique - Hôpitaux de Paris

**Verification Date June 2015**
